# Delayed Diagnosis of Psoriatic Arthritis Mutilans due to Arthritis Prior to Skin Lesion

**DOI:** 10.1155/2018/4216938

**Published:** 2018-11-04

**Authors:** Takeshi Mochizuki, Katsunori Ikari, Ken Okazaki

**Affiliations:** ^1^Department of Orthopedic Surgery, Kamagaya General Hospital, Chiba, Japan; ^2^Department of Orthopedic Surgery, Tokyo Women's Medical University, Tokyo, Japan

## Abstract

Psoriatic arthritis (PsA) is a type of inflammatory arthritis characterized by cutaneous psoriasis, peripheral joint damage, axial joint damage, and enthesitis and is usually diagnosed after the appearance of psoriatic skin disease. PsA mutilans is relatively rare in Japan. In the present case, the patient was diagnosed with PsA with foot mutilans deformity only. Hand and spinal radiograph findings were unremarkable. As in the present case arthritis is occurred prior to the onset of skin lesion, we should make early diagnosis to prevent mutilans deformity.

## 1. Introduction

Psoriatic arthritis (PsA) is a type of inflammatory arthritis characterized by cutaneous psoriasis, peripheral joint damage, axial joint damage, and enthesitis and is usually diagnosed after the appearance of psoriatic skin disease. The prevalence of psoriasis in adults ranges from 0.91% (United States) to 8.5% (Norway) [1]. At 0.34%, the prevalence of psoriasis is lower in Japan than in Europe and the United States [2]. The prevalence of PsA in Japan is 0.001–0.03% in the general population [3]. In contrast, the prevalence of PsA was reported to be 14.3% (range, 8.8–20.4%) among 3,021 Japanese patients with psoriasis between March 2003 and February 2014 in three major Japanese areas; this includes the following types of PsA: distal interphalangeal (DIP) type (8.9%), oligoarthritis type (28.6%), polyarthritis type (60.4%), mutilans type (0.5%), and no peripheral arthritis (0.7%) [4]. In a multicenter study conducted by the Japanese Society for Psoriasis Research, types of PsA among 1,282 newly diagnosed patients were as follows: polyarthritis type (36%), DIP type (26%), oligoarthritis type (22%), spondylitis type (8.1%), mutilans type (1.8%), and unknown (6.1%) according to the Moll and Wright criteria [5].

As mentioned above, PsA mutilans is relatively rare in Japan. In the present case, the patient was diagnosed with PsA with foot mutilans deformity only. We report this case for the purpose of education.

## 2. Case Report

**Figure 1:**
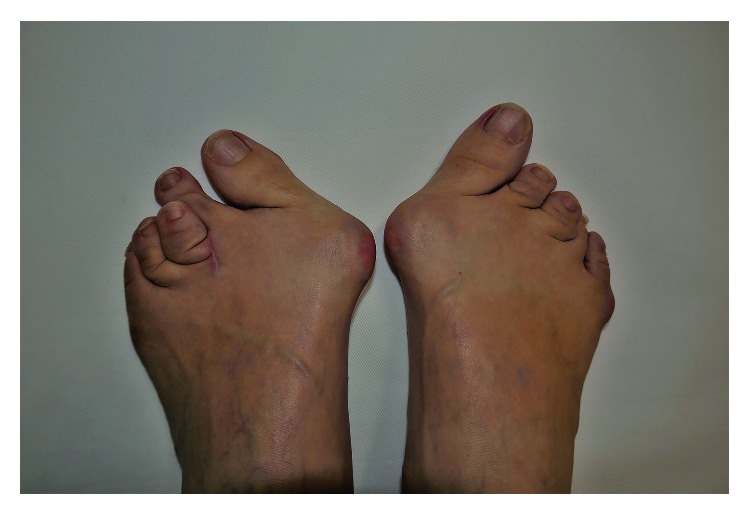
Deformities involving the toes on both feet.

**Figure 2:**
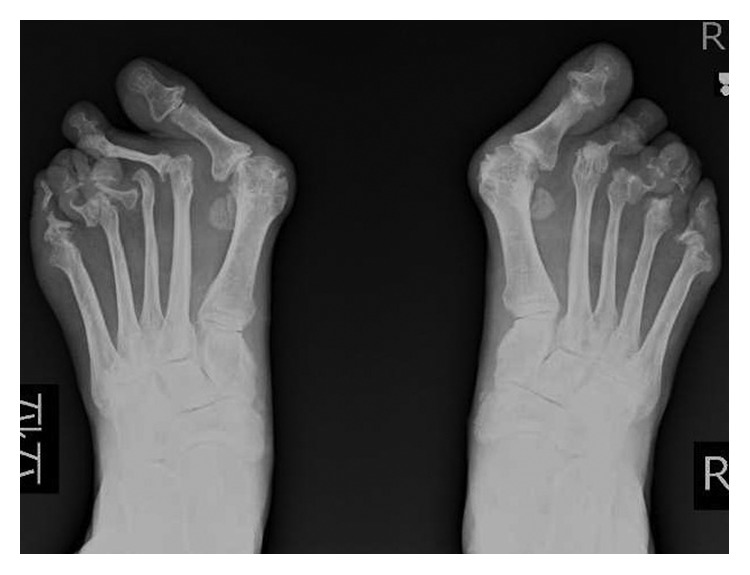
Plain radiographs of the feet.

**Figure 3:**
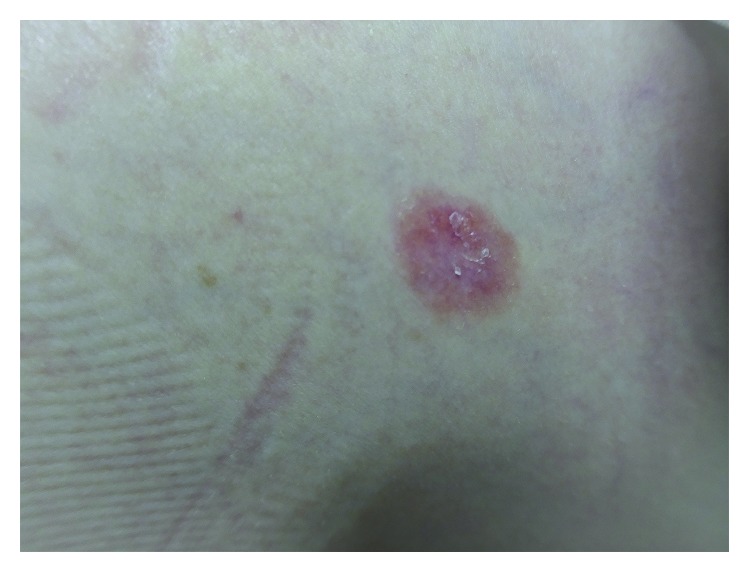
The rash on the chest.

**Figure 4:**
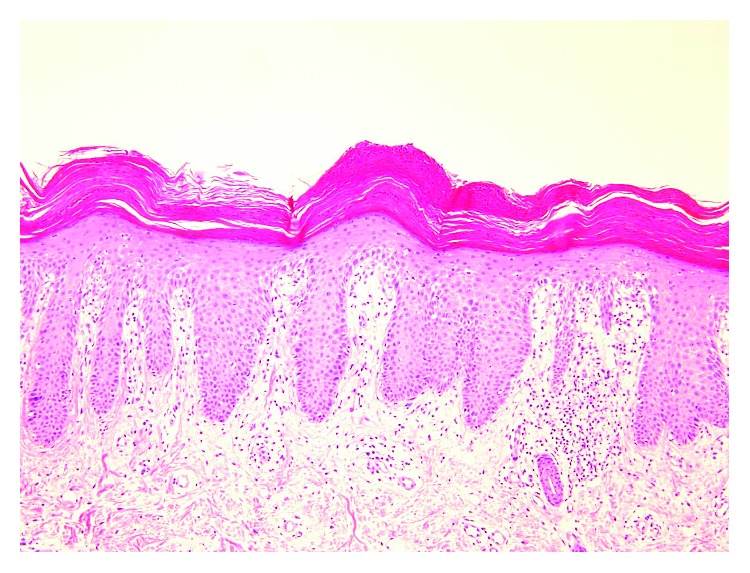
Histologic findings (hematoxylin-eosin (HE), ×100).

A 39-year-old female presented to our orthopaedic clinic with plantar pain and a gait disturbance and deformities involving the toes on both feet (Figure 1). One decade ago, she was examined and suspected of rheumatoid arthritis by several orthopaedic surgeons, but she has not been diagnosed a definitive diagnosis and prescribed nonsteroidal anti-inflammatory drugs. Plain radiographs of the feet showed severe joint destruction in the proximal interphalangeal (PIP) joints of the lesser toes, with joint space widening and digit shortening consistent with arthritis mutilans (Figure 2). Hand and spinal radiograph findings were unremarkable. Rheumatoid factor and anticyclic citrullinated peptides antibody were negative, and the C-reactive protein level was normal (0.10 mg/dL). She has no family history of psoriasis, PsA, and rheumatic diseases. Although no skin irregularities were observed on the feet, a rash was noted on the chest (Figure 3). Because PsA was suspected, a skin biopsy of the chest was obtained that showed parakeratosis, hyperkeratosis, and regular acanthosis. Histologic findings were consistent with psoriasis (Figure 4). From the results, she diagnosed PsA with mutilans deformity. After treatment with adalimumab, the skin rash resolved and the pain was relieved.

Written informed consent was obtained from the patient.

## 3. Discussion and Conclusions

The present case was diagnosed as PsA mutilans with feet deformities only and was diagnosed late. PsA mutilans is relatively rare in Japan. The Classification criteria for psoriatic arthritis (CASPAR) are widely used for diagnosis of PsA (sensitivity, 91.4%; specificity, 98.7%) [6]. In short, this system identifies PsA based on the presence of ≥3 points by scoring for current evidence of psoriasis, 2 points; and a personal history or a family history of psoriasis, typical psoriatic nail, a negative test result for the presence of rheumatoid factor, current dactylitis, or radiographic evidence of juxta-articular new bone formation, 1 point each. The present case was assigned a score of 4 by CASPAR criteria.

A case of PsA mutilans type with arthritis has been previously reported in a patient with rheumatoid arthritis [7]. One of the clinical manifestations of this condition is shortening of one or more digits due to severe osteolysis, a deformity called “opera glass finger” or “telescoping finger.” The radiographic findings of PsA mutilans suggest gross osteolysis and pencil-in-cup deformities in joints, as well as rapid progress. Radiographic features in PsA mutilans include bone resorption (41%), joint ankylosis (21%), pencil-in-cup changes (16%), total joint erosion (14%), and joint subluxation (7%) [8]. In the present case, severe progression of bone resorption, joint erosion, and joint subluxation were observed in the feet, particularly in the PIP joints. As a result, the present patient experienced a gait disturbance due to joint destruction of the feet.

Early diagnosis and treatment is important to prevent progression of joint destruction. A delay of ≥6 months from symptom onset to the first visit with a rheumatologist contributes to the development of peripheral joint erosions and worse long-term physical function [9]. Psoriasis has been reported to occur prior to the onset of arthritis in 76.2% of patients, with arthritis occurring 11.2 years after psoriasis [5]. Therefore, it is necessary to investigate skin lesions for early diagnosis of psoriasis. Psoriasis typically occurs on the skin of the scalp, knees, elbows, and lower back. In a Japanese survey, the most common skin lesions among patients <65 years old were the knees (10.8%), elbows (10.6%), back (9.1%), scalp (8.1%), and chest (7.1%) [10]. In the present case, we suspect that the primary skin lesion was on the chest, therefore leading to a delay in the diagnosis of PsA.

In conclusion, PsA mutilans is relatively rare. We have presented a case in which diagnosis of PsA mutilans was delayed and led to dysfunction in daily life. As in the present case arthritis is occurred prior to the onset of skin lesion, we should make early diagnosis to prevent mutilans deformity.
